# Severe acute respiratory syndrome coronavirus-2 medical solid waste treatment: A need for efficient and effective strategies in low-resourced settings

**DOI:** 10.1177/0734242X21998739

**Published:** 2021-06

**Authors:** Saverio Bellizzi, Paola Murgia, Antonella Angioi, Gualtiero Canu, Catello M Panu Napodano, Antonio Manca

**Affiliations:** 1 Medical Epidemiologist, Independent Consultant, Geneva, Switzerland; 2 University of Sassari, Sassari, Sardegna, Italy; 3 Polo Ospedaliero San Francesco, ASSLL, Nuoro, Italy; 4 Candiolo Cancer Institute FPO, IRCCS, Candiolo, Turin, Italy

**Keywords:** Severe acute respiratory syndrome coronavirus-2, relative humidity, air temperature, ultraviolet irradiation, waste management, medical disposal

## Abstract

Understanding infections related to handling healthcare waste products is of critical importance and the application of simple and low-cost strategies remain a priority in low-income and middle-income countries to protect healthcare workers. We examined the potential effect of relative humidity (RH), air temperature and ultraviolet irradiation (UI) to establish an efficient and effective way to facilitate disposal of medical waste. Literature is emerging on the effect of high RH and high temperature, which would increase airborne mass deposition and decrease the viability of viruses in both airborne particles and on surfaces. On the other hand, severe acute respiratory syndrome coronavirus-2 has been proven to be susceptible to UI when suspended in air like other coronaviruses. An innovative approach utilizing environmental conditions might represent an effective and efficient way to ensure better and sustainable protection of the healthcare workers in low-resourced settings.

## Introduction

A very recent study conducted by the International Council of Nurses confirmed that around 1500 nurses have died in 44 countries because of severe acute respiratory syndrome (SARS) coronavirus-2 infection and that fatalities could be up to 20,000 among healthcare workers globally (International Council of Nurses, 2020).

Understanding infections related to handling healthcare waste products is of critical importance and the application of simple and low-cost strategies remain a priority in low-income and middle-income countries to protect healthcare workers. This becomes even more relevant in settings with no standardized medical solid waste management scheme.

Specifically, biomedical wastes are hazardous since they may host potential virus components beneath human tissues, items contaminated with blood bags as well as with other items such as needles, syringes and body fluids. Therefore, infected waste is assumed to harbour pathogenic organisms, which in turn can trigger disease in susceptible hosts.

The survival of the SARS coronavirus-2 on surfaces was subject to some research, however these pieces of research were not conclusive (Ugom, 2020).

Medical wastes including personal protective equipment such as masks, gloves and ventilators, need to be discarded correctly to avoid community spreading of the coronavirus disease. In the case of the current SARS coronavirus-2 pandemic, masks and gloves represent an enormous quantity of medical wastes and are likely to be improperly disposed of in many low-income and middle-income countries. Also, what is classified as medical waste really depends on the location the waste is coming from (Ugom, 2020).

The purpose of this short communication is to explore the role of different environmental aspects that could play a major role on the survival of SARS coronavirus-2 on surfaces, thus providing key insights to establish an efficient and effective way to facilitate disposal.

Specifically, we examined the potential effect of relative humidity (RH), air temperature (AT) and ultraviolet irradiation (UI).

## Methods

We searched MEDLINE Ovid and Scopus databases for relevant articles. The search terms included: “Mask”; “Facemask”; “Respirator”; “Surgical mask”; “Medical mask”; “Personal protective equipment”; “Personal protective clothing”; “Decontamination”; “COVID-19”; “SARS-CoV-2”; “SARS coronavirus-2”; “Relative Humidity”; “RH”; “Air Temperature”; “AT”; “Ultraviolet Irradiation”; “UI”; and “Waste Management”. We also searched for unpublished manuscripts in medRxiv and bioRxiv, as well as news and reports using Google. The search was last updated on 20 November 2020. Only articles published in English were considered for this review.

The criteria for selecting reports included the presence of SARS-coronavirus-2 or other closely related coronaviruses (e.g., SARS-CoV-1) and the exposures of interest whether measured directly or inferred: RH; AT; and UI. Epidemiological studies of any health outcome and of any study population as well as of any design, including cross-sectional, case–control and cohort studies, were considered. We excluded studies that did not include measurement as well as purely methodological analysis. Two reviewers (SB and AM) evaluated the eligibility of studies. In case of discrepancy a third reviewer (CMPN) provided arbitration.

The initial search provided 54 non-duplicate records, of which 49 full texts were assessed for eligibility. After exclusion of 11 records that did not meet the pre-established inclusion criteria, 38 studies were retained for qualitative synthesis. Of these 38 studies, 16 dealt only with levels of exposure and 22 combined exposure and outcomes; among the latter, nine studies reported findings measured by quantitative methods.

## RH

As per the literature, several viral respiratory diseases such as influenza and SARS coronavirus-2 itself benefit from high RH to spread, suggesting that suspension of a sufficient quantity of droplets in the air is more important in terms of infectivity when compared to the adverse effect of dry air on the human immune system (Chen et al., 2020).

However, several studies have detected how RH was inversely related to daily deaths from COVID-19 (Ma et al., 2020). Chan et al. (2011) have also demonstrated how despite this SARS coronavirus-2 could survive for over 5 days on smooth surfaces, and its viability reduced rapidly when the temperature or RH increased. Therefore, SARS coronavirus-2 may be less stable in high-humidity environments.

On the other hand, evidence of rapid transmission in the African equatorial and Amazon rainforest regions suggests that the effect of temperature and humidity on SARS coronavirus-2 transmission is not sufficient to fully inhibit the pandemic (Wu et al., 2020).

Based on the available data, we can say that high RH and high temperature appear to increase airborne mass deposition and decrease the viability of viruses in both airborne particles and on surfaces. Although higher humidity may increase the atmospheric suspended matter, the quantity of virus deposited on surfaces, and virus survival time in droplets on surfaces, the reduction of the virus spread by indirect air transmission may be an important factor behind the reduced spreading of SARS coronavirus-2 in a humid climate (Mecenas et al., 2020).

However, any decrease in viability does not alleviate the need to maintain physical distancing, as well as adequate cleaning, disinfection, and ventilation.

## AT

There seems to be no doubt on the role of temperature in changing the SARS coronavirus-2 human-to-human transmission. Specifically, various reports suggest that there might be an optimal temperature for the viral transmission, with low temperatures significantly contributing to the viability, transmission rate, and survival of coronaviruses (thus suggesting that colder regions in the world should adopt the strictest social control measures) (Wang et al., 2020).

A very recent systematic review has shown that the spread of SARS coronavirus-2 seems to be lower in warm and wet climates. However, the level of evidence was rated low, as temperature and humidity alone do not explain most of the variability of the SARS coronavirus-2 outbreak (Mecenas et al., 2020).

As proposed by Mecenas et al. (2020), similarly to what occurs with the predecessor of the new coronavirus, heat intolerance of the virus is probably related to the breakdown of their lipid bilayer.

Certain clinical tests found that the infection rate for some seasonal airborne viruses was reduced to zero at temperature 30°C at a certain humidity level. On the other hand, a recent study has shown that the vulnerability to SARS coronavirus-2 is reduced drastically even at 27°C, without considering any effect of humidity. In addition to that, when the temperature was above 32°C, an unusually low number of the reported cases, as well as deaths, was observed (Roy, 2020).

## UI

The UI is increasingly used as an alternative to other methods such as chlorination because of its high efficiency in disinfection for microorganisms (viruses and bacteria). The popularity of UI technology is mainly due to its low costs and the lack of toxic residues.

The integrated UI approach has been found to be a very suitable alternative for disinfecting SARS coronavirus-2 medical waste products and this finding is consistent with prior studies on SARS-CoV-1 (Belhadi et al., 2020).

The SARS coronavirus-2 is relatively easily inactivated by UI-C light and when aerosolized the virus is likely to have a UI susceptibility constant, *Z*_ur_, that is similar to that exhibited by other coronaviruses in air. This suggests that SARS coronavirus-2 when suspended in air should be reasonably easy to inactivate using UI light at 254 nm.

Using published data from various sources, it is shown that the SARS coronavirus-2, the causative agent of COVID-19, is highly likely to be susceptible to UI-C damage when suspended in air, with a UI susceptibility constant likely to be in the region 0.377–0.590 m^2^ J^−1^, similar to that for other aerosolized coronaviruses (Beggs et al., 2020).

## Conclusions

The majority of research in the area of occupational health and the management of research so far have been concentrated on high-resourced settings with schemes and systems where the management of hazardous waste and its regulations is well established. This is not the case for many low-resourced settings where standardized strategies for disposal and management of medical waste are not in place, while being often mixed with municipal waste.

Efficient and effective technologies for virus inactivation could be obtained making the best use of natural sources of RH, AT and UI. This could be especially true in countries where abundance of solar radiation and long sunny hours may help to achieve such “virus-inactivating” conditions. Furthermore, the proposed technology presented depends on producing a simple collection system based on minimum protection procedures and low costing.

A temperature above 40°C× can be obtained in summer in many developing countries in Sub-Saharan Africa as well as in the Middle East and in South-Eastern Asia where sunny hours per year can exceed 40°C. Similarly, high RH is present in such countries during several months throughout the year.

The strengths of our review include a detailed search of not only several databases of scientific publications, but also of national and agency reports and other grey literature to identify eligible studies, and an evaluation of all included studies using a rigorous method. A limitation of this review is that we were not able to quantitively assess the risk of publication bias due to the paucity of evidence.

While the presented findings mostly relate to a non-specific sector, we would strongly suggest conducting similar experimental analyses in the health-sector environment with clear research questions related to minimizing the risk of infection among individuals handling medical waste products.

This innovative approach utilizing environmental conditions might represent a turning-point to ensure effective treatment of medical waste generated from the management of COVID-19 patients with minimal cost, thus contributing to better and sustainable protection of the healthcare workers in several developing countries.
